# LncRNA AK020546 protects against cardiac ischemia–reperfusion injury by sponging miR-350-3p

**DOI:** 10.18632/aging.203038

**Published:** 2021-05-13

**Authors:** Meiqi Zhang, Kang Cheng, Huan Chen, Jianfeng Tu, Ye Shen, Lingxiao Pang, Weihua Wu, Zhenfei Yu

**Affiliations:** 1 Department of Intensive Care Unit, Hangzhou Hospital of Traditional Chinese Medicine (Dingqiao), Guangxing Affiliated Hospital of Zhejiang Chinese Medical University, Hangzhou, Zhejiang, China; 2 Department of Emergency Medicine, Zhejiang Provincial Peoples Hospital, People’s Hospital of Hangzhou Medical College, Hangzhou, Zhejiang, China

**Keywords:** lncRNA, cardiac ischemia reperfusion, miR-350-3p

## Abstract

Long non-coding RNAs (lncRNAs) have been implicated in the development of cardiovascular diseases. We observed that lncRNA AK020546 was downregulated following ischemia/reperfusion injury to the myocardium and following H_2_O_2_ treatment in H9c2 cardiomyocytes. *In vivo* and *in vitro* studies showed that AK020546 overexpression attenuated the size of the ischemic area, reduced apoptosis among H9c2 cardiomyocytes, and suppressed the release of reactive oxygen species, lactic acid dehydrogenase, and malondialdehyde. AK020546 served as a competing endogenous RNA for miR-350-3p and activated the miR-350-3p target gene *ErbB3*. MiR-350-3p overexpression reversed the effects of AK020546 on oxidative stress injury and apoptosis in H9c2 cardiomyocytes. Moreover, *ErbB3* knockdown alleviated the effects of AK020546 on the expression of ErbB3, Bcl-2, phosphorylated AKT, cleaved Caspase 3, and phosphorylated Bad. These findings suggest lncRNA AK020546 protects against ischemia/reperfusion and oxidative stress injury by sequestering miR-350-3p and activating ErbB3, which highlights its potential as a therapeutic target for ischemic heart diseases.

## INTRODUCTION

Acute myocardial infarction (AMI), characterized by inadequate blood flow and oxygen supply to the heart due to blocked arteries, is the leading cause of morbidity and mortality worldwide [1]. The most effective therapeutic approach to protect the heart against AMI is to restore its blood supply [2]. However, reperfusion and reoxygenation exacerbate tissue injury as well as induce inflammatory response [3]. One of the major factors contributing to I/R injury is apoptosis induced by mitochondrial dysfunction and increased lipid peroxides [4].

Non-coding RNAs (ncRNAs) have been implicated in cardiovascular cell signaling and pathogenesis of several cardiac diseases [5, 6]. Long non-coding RNAs (lncRNAs) are a family of ncRNAs with the length of more than 200 nucleotides [7]. LncRNAs participate in the regulation of gene expression at epigenetic, transcriptional, and post-transcriptional levels. They have been indicated to play critical role in the cardiac remodeling, myocardial hypertrophy, and cardiac I/R injury [8–10]. For instance, lncRNA TUG1 knockdown inhibits I/R-induced myocardial injury by altering miR-142-3p expression and autophagy [11]. Similarly, lncRNA H19 ameliorates cardiac I/R injury by targeting miR-22-3p [12]. LncRNA CAIF blocks p53-induced myocardin transcription, further preventing autophagy-related myocardial infarction [13].

LncRNAs participate in the progression of AMI by sponging microRNAs (miRNAs). The expression of several miRNAs is induced by oxidative stress. For example, tomato supplementation protected against oxidative stress and left ventricular mass and restored heart function by downregulating miR-350 [14]. Moreover, overexpression of miR-350 has been reported to contribute to heart hypertrophy by inactivating JNK and p38 pathways [15].

Erb-b2 receptor tyrosine kinase 3 (ErbB3), come from epidermal growth factor receptor tyrosine kinase (RTK) family [16], is a ligand activated by neuregulin genes [17]. ErbB3 is required for normal cerebellar and cardiac development and its activation improves the formation of heart valve mesenchyme [18, 19]. Binding of NRG1 to ErbB3 activates downstream signaling molecules, including PI3K/AKT, Ras/ERK, and Src/FAK. For instance, ErbB3 enhanced the proliferation and survival of normal human cardiac ventricular fibroblasts by activating PI3K/AKT signaling [20]. Moreover, ErbB3 suppresses mitochondrial dysfunction and apoptosis of cardiomyocytes [21].

In the present work, we studied the function of lncRNA AK020546 in cardiac ischemic injury. Functional studies revealed that AK020546 reduced the infarct size and myocardial cell apoptosis, and decreased H_2_O_2_-induced apoptosis of H9c2 cardiomyocytes. In addition, it binds to and sequesters miR-350-3p to increase the expression of its downstream target gene *ErbB3*. These findings highlight the function of lncRNA AK020546 in maintaining cardiovascular health and provide potential therapeutic targets for cardiac ischemic injury.

## RESULTS

### LncRNA AK020546 was downregulated in I/R injury

**Figure 1:**
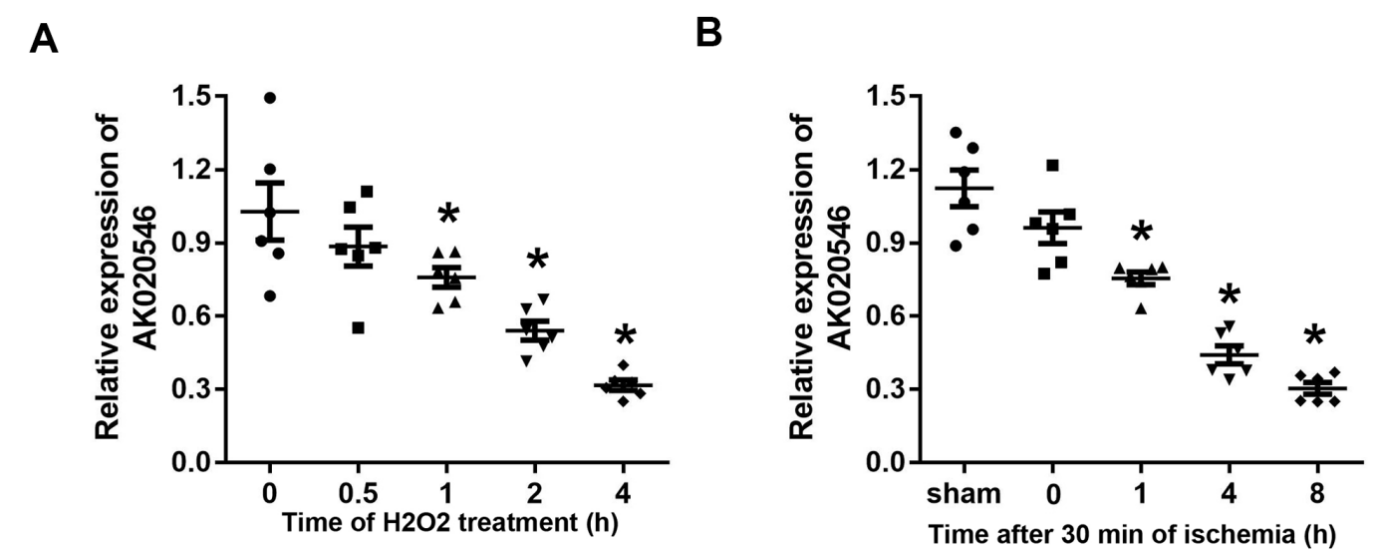
**LncRNA AK020546 was downregulated in H9c2 cardiomyocytes and myocardium subjected to H_2_O_2_ treatment and I/R injury.** (**A**) The expression of lncRNA AK020546 in H9c2 cardiomyocytes at different time points after H_2_O_2_ treatment was detected with qPCR (*n* = 5). (**B**) The expression of lncRNA AK020546 in the myocardium at different time points of reperfusion was evaluated by qPCR (*n* = 5). ^*^p < 0.05 vs oh or sham group.

To assess the function of AK020546 in I/R injury, we established an oxidative stress model using H9c2 cells and an I/R model in rats and studied its expression. The results indicated that AK020546 expression was notably reduced in H9c2 cells 1 h after H_2_O_2_ treatment and in the myocardium 1 h after ischemia (Figure 1A, 1B).

### Overexpression of lncRNA AK020546 attenuated H_2_O_2_-induced apoptosis and oxidative stress in H9c2 cardiomyocytes

**Figure 2:**
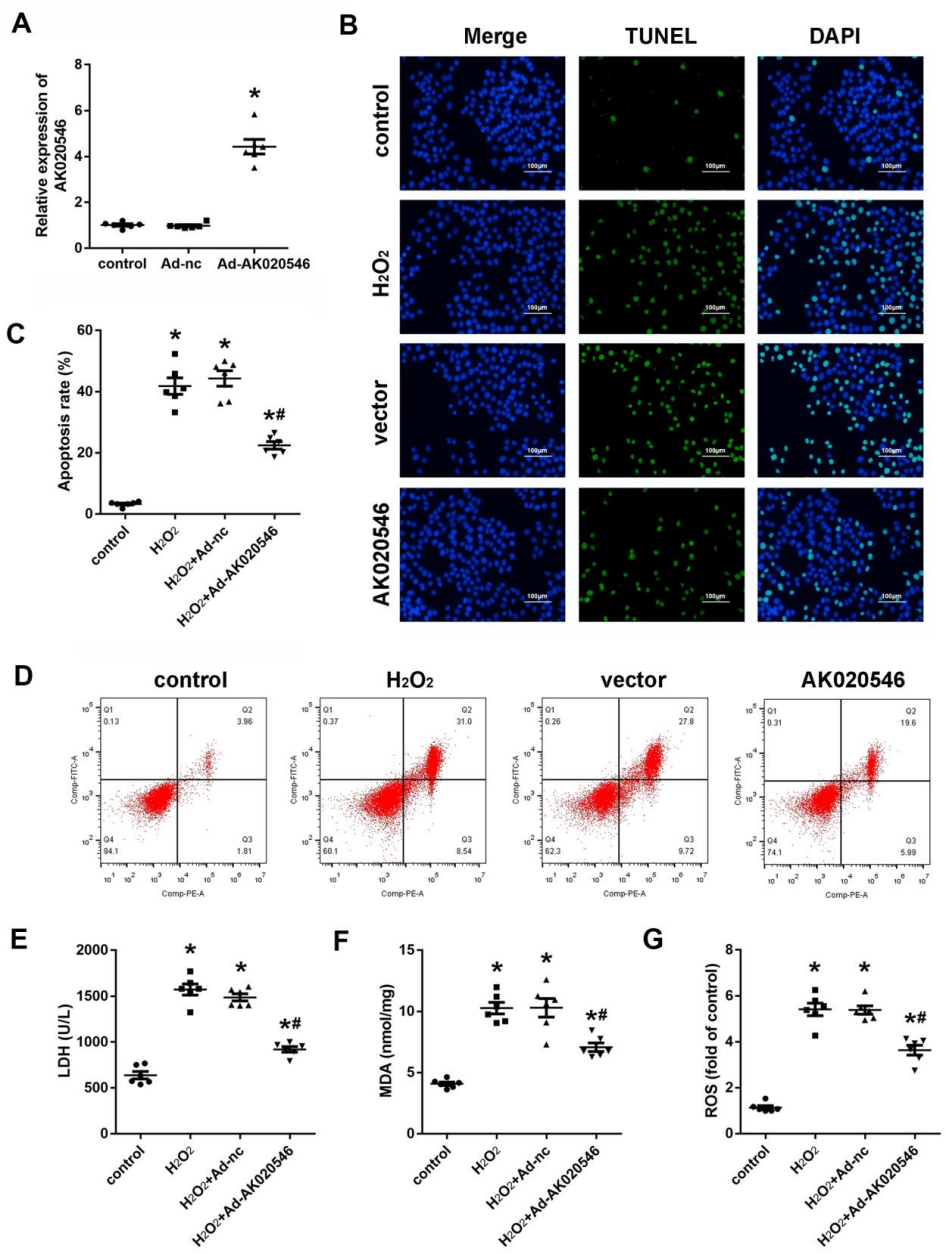
**lncRNA AK020546 inhibited I/R-induced oxidative stress and apoptosis *in vitro*.** (**A**) qPCR verified the lncRNA AK020546-overexpressing efficiency of adenovirus particles in H9c2 cardiomyocytes (*n* = 5). (**B**) TUNEL staining was used to detect the apoptosis of H9c2 cardiomyocytes (*n* = 5). (**C**, **D**) Flow cytometry and Annexin V/propidium iodide (PI) staining were carried out to detect H9c2 cardiomyocyte apoptosis (*n* = 5). (**E**–**G**) The levels of oxidative markers, such as LDH, MDA, and ROS, were detected using commercial kits (*n* = 5). ^*^p < 0.05 versus Ad-nc or control group, ^#^*p* < 0.05 vs H_2_O_2_ + Ad-nc or control group.

Because AK020546 was downregulated in I/R injury-exposed myocardium and H_2_O_2_-treated H9c2 cardiomyocytes, we speculated that it participated in the progression of I/R injury. First, we established an *in vitro* model of oxidative stress injury using H_2_O_2_ in H9c2 cardiomyocytes. Quantitative realtime PCR (qPCR) analysis revealed a good efficiency of AK02054-overexpressing adenovirus (Figure 2A). Terminal deoxynucleotidyl transferase dUTP nick end labeling (TUNEL) results revealed that AK020546 reduced H_2_O_2_-induced apoptosis of H9c2 cardiomyocytes (Figure 2B). Flow cytometry revealed similar results (Figure 2C, 2D). Because H9c2 cardiomyocyte apoptosis is linked to oxidative stress, we next evaluated the change in the levels of malondialdehyde (MDA), lactic acid dehydrogenase (LDH), and reactive oxygen species (ROS) in H9c2 cardiomyocytes. The results indicated that AK020546 overexpression remarkably reversed H_2_O_2_-induced elevated levels of MDA, LDH, and ROS (Figure 2E–2G).

### LncRNA AK020546 overexpression inhibited cardiac I/R injury *in vivo*

**Figure 3:**
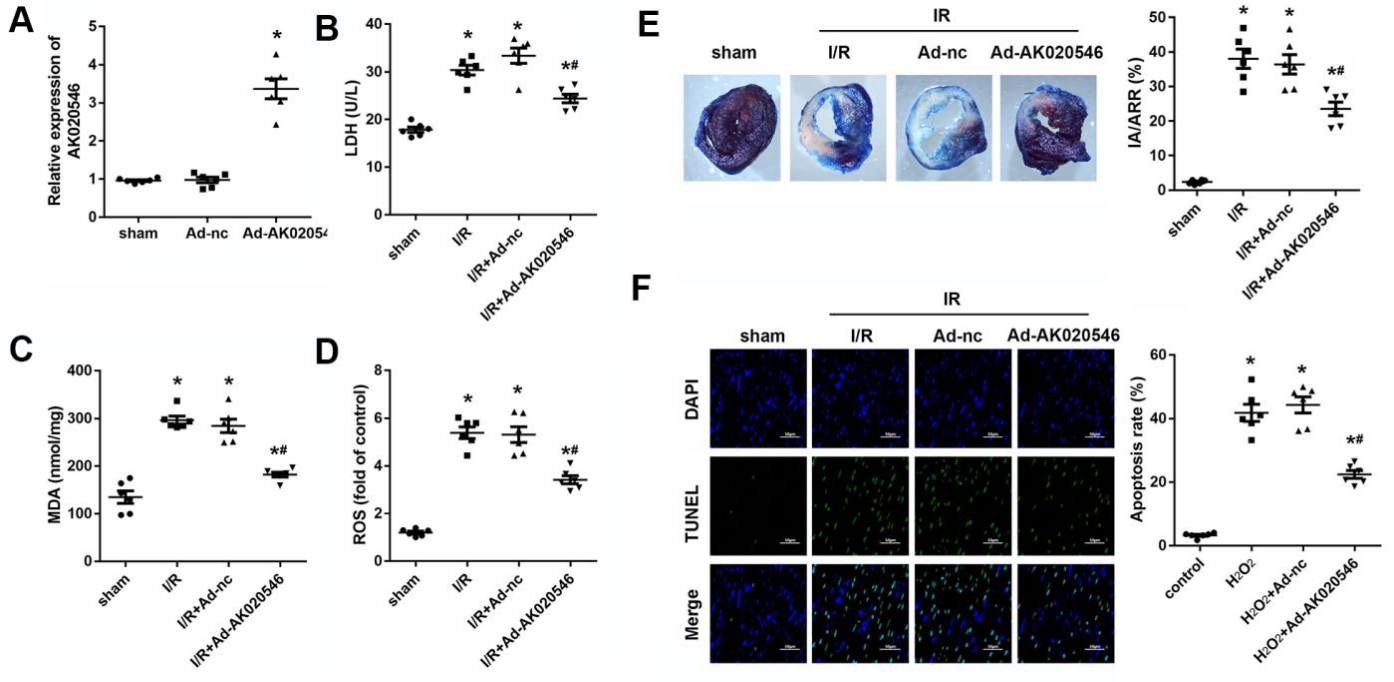
**LncRNA AK020546 inhibited I/R injury-induced oxidative stress and apoptosis *in vivo*.** (**A**) qPCR confirmed the lncRNA AK020546-overexpressing efficiency of adenovirus particles in the myocardium in comparison with the control group (*n* = 8). (**B**–**D**) The levels of oxidative markers, such as LDH, MDA, and ROS, in the myocardium were detected using commercial kits (*n* = 8). (**E**) TTC staining was performed to evaluate the infarct area of the heart (*n* = 8). (**F**) TUNEL staining and α-smooth muscle actin (SMA) staining were performed to detect the apoptosis of specific H9c2 cardiomyocytes (*n* = 8). ^*^p < 0.05 vs Ad-nc or sham group, ^#^*p* < 0.05 vs I/R + Ad-nc or control group.

**Figure 4:**
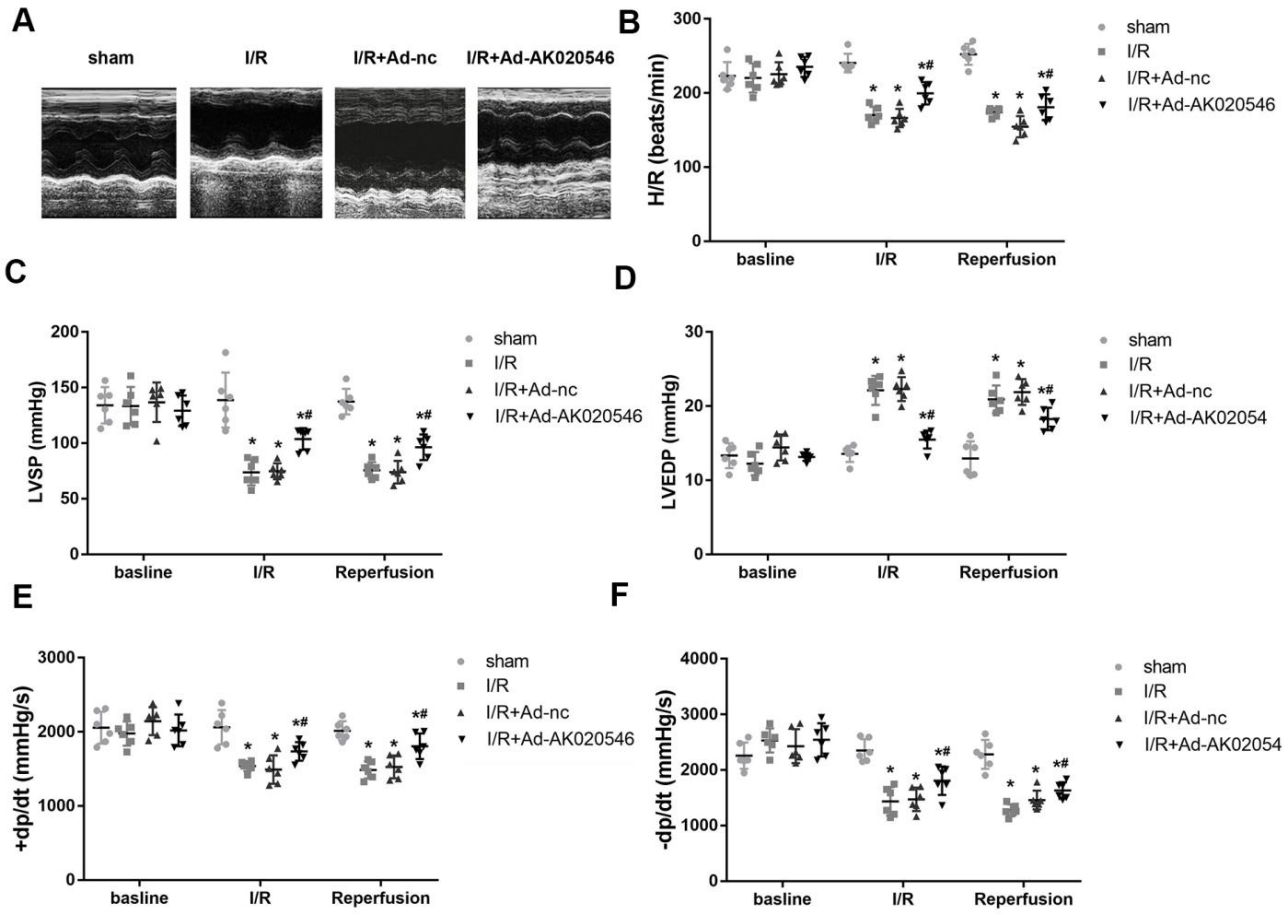
**LncRNA AK020546 alleviated I/R injury-induced cardiac dysfunction *in vivo*.** (**A**–**F**) Echocardiographic parameters of rats including LVSP, LVEDP, ±dp/dt, and heart rate were evaluated with an ultrasound device (*n* = 8). ^*^p < 0.05 vs sham group, ^#^*p* < 0.05 vs I/R + Ad-nc or control group.

To further confirm the functions of AK020546 in I/R injury, we constructed an I/R model in rats. Figure 3A shows increased expression of AK020546 in the myocardium following its overexpression using an adenovirus construct. We next conducted *in vitro* studies that showed that AK020546 overexpression reduced the elevated levels of MDA, LDH, and ROS (Figure 3B–3D). Furthermore, compared with the Ad-control (Ad-nc) group, 2,3,5-triphenyltetrazolium chloride (TTC) staining revealed that AK020546 overexpression reduced I/R injury-induced infarct area. The reduction in the infarct size was 28.45 ± 14.64% (Figure 3E). TUNEL assay was conducted to evaluate apoptosis (Figure 3F). We next investigated cardiac function, a parameter impaired by I/R injury. The results indicated that AK020546 elevated left ventricular systolic pressure (LVSP) and maximal LV contractility rate (±dp/dt); however, it decreased left ventricular end-systolic diameter (LVEDP). Moreover, AK020546 overexpression increased the heart rate during both I/R injury and reperfusion (Figure 4A–4F).

### LncRNA AK020546 sponged miR-350-3p

**Figure 5:**
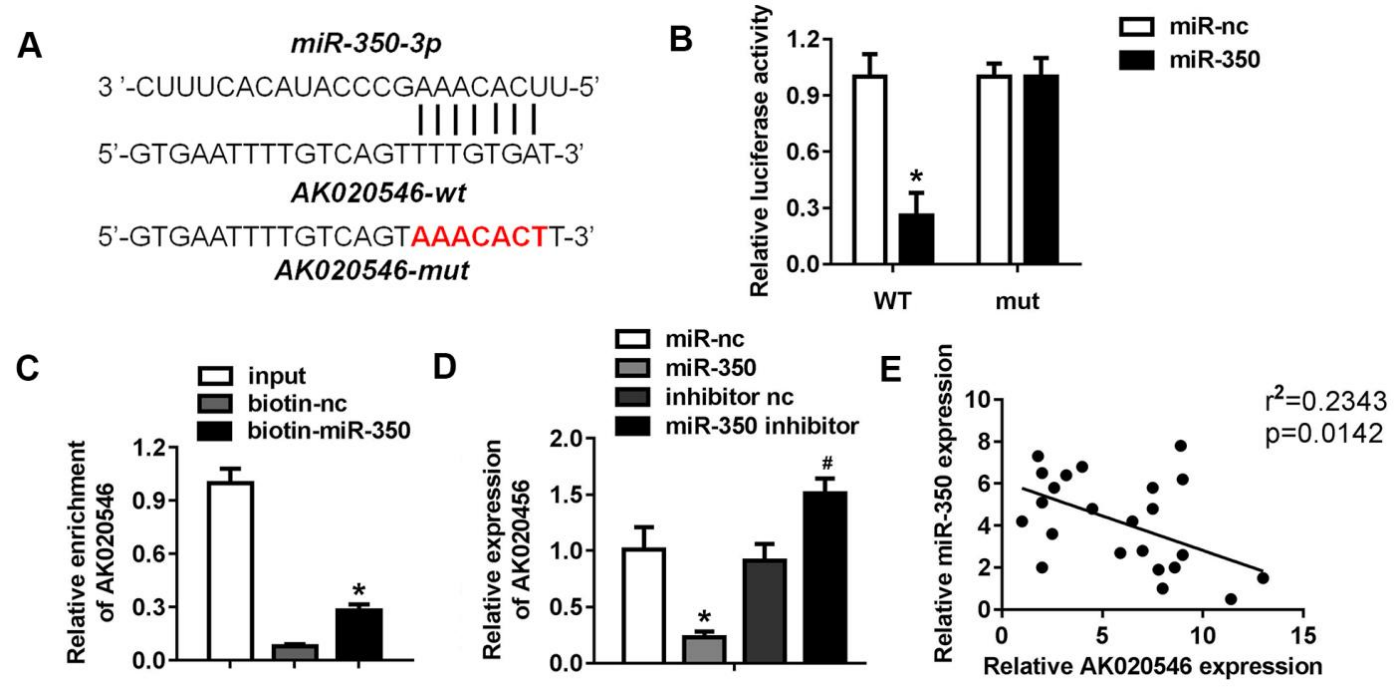
**LncRNA AK020546 sponged miR-350-3p in H9c2 cardiomyocytes.** (**A**) The potential targeting region predicted by bioinformatics analysis. (**B**) Luciferase assay was performed to verify whether miR-350-3p targeted AK020546 in H9c2 cardiomyocytes (*n* = 5). (**C**) RNA pull-down assay confirmed the interaction between AK020546 and miR-350-3p (*n* = 5). (**D**) qPCR was used to detect the expression of AK020546 in different groups (*n* = 5). (**E**) Pearson’s analysis was performed to investigate the correlation between AK020546 and miR-350-3p (*n* = 5). ^*^p < 0.05 versus miR-nc or biotin-nc, ^#^*p* < 0.05 vs inhibitor nc group.

LncRNAs function as competing endogenous RNAs and sponge target miRNAs and subsequently abrogate their functions. Therefore, we next explored whether AK020546 sponged miRNAs. MiRDB, an online database for predicting target miRNAs, predicted miR-350-3p as a potential target of AK020546. Figure 5A shows the target region between AK020546 and miR-350-3p. We found that miR-350-3p downregulated the luciferase activity in H9c2 cardiomyocytes co-transfected with pGL3-wt-lncRNA AK020546, but not the pGL3-mut-lncRNA AK020546 (Figure 5B). RNA pull-down assay showed that AK020546 could directly bind to miR-350-3p endogenously. We found that the biotin-labeled miR-350-3p probe enriched the levels of AK020546, indicating the binding between lncRNA AK020546 and miR-350-3p (Figure 5C). Moreover, qPCR results confirmed that miR-350-3p overexpression reduced the level of AK020546, whereas its knockdown reversed this effect (Figure 5D). Pearson’s analysis indicated an inverse relation between lncRNA AK020546 and miR-350-3p (Figure 5E).

### MiR-350-3p reversed the effect of lncRNA AK020546-induced apoptosis of H9c2 cardiomyocytes

**Figure 6:**
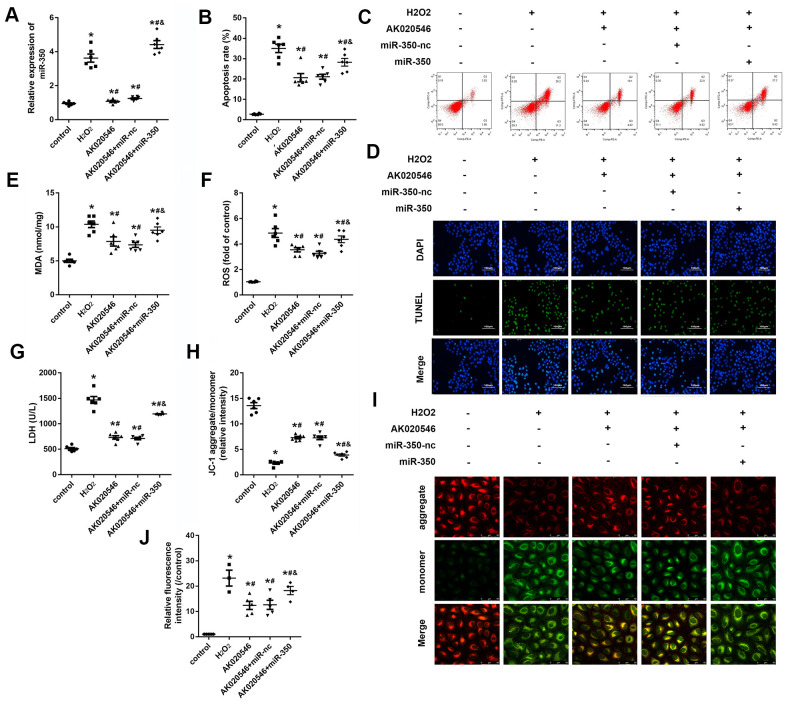
**MiR-350-3p reversed the effect of lncRNA AK020546 on cardiac I/R injury *in vitro*.** (**A**, **B**) qPCR was used to assess the level of miR-350-3p in H9c2 cardiomyocytes under different treatments (*n* = 5). (**B**, **C**) Flow cytometry and Annexin V/propidium iodide (PI) staining were performed to detect the apoptosis of H9c2 cardiomyocyte (*n* = 5). (**D**) TUNEL staining was performed to detect the apoptosis of specific H9c2 cardiomyocytes (*n* = 5). (**E**–**G**) The levels of oxidative markers such as LDH, MDA, and ROS were assessed with commercial kits (*n* = 5). (**H**, **I**) JC-1 staining was performed to evaluate the mitochondrial membrane potential (*n* = 5). (**J**) MitoSOX staining was used to evaluate the mitochondrial ROS. ^*^p < 0.05 vs control, ^#^*p* < 0.05 vs H_2_O_2_ group, ^&^*p* < 0.05 vs AK020546 + miR-nc group.

LncRNA AK020546 works as a sponge of miR-350-3p in H9c2 cardiomyocytes. To investigate the function of miR-350-3p in I/R injury, we conducted rescue experiments. H9c2 cardiomyocytes were co-transfected with adenovirus particles overexpressing AK020546 and miR-350-3p mimic along with their negative controls. We found that H_2_O_2_ promoted the expression of miR-350-3p, whereas AK020546 overexpression reversed this effect. Co-transfection with AK020546 and miR-350-3p elevated the expression of AK020546 in comparison to AK020546 transfection alone (Figure 6A). TUNEL assay and flow cytometry revealed that AK020546 reduced H_2_O_2_-induced apoptosis. MiR-350-3p remarkably reversed this alteration (Figure 6B–6D). Furthermore, miR-350-3p reversed AK020546-induced release of MDA, LDH, and ROS (Figure 6E–6G). 5, 5’, 6, 6’-tetrachloro-1, 1’, 3, 3’-tetraethyl-imidacarbocyanine iodide (JC-1) staining revealed that AK020546 overexpression elevated the levels of mitochondrial membrane potential (MMP) (reduced by H_2_O_2_), whereas miR-350-3p reversed this effect (Figure 6H, 6I). Mitochondrial ROS was detected using MitoSOX staining. The results indicated that H2O2 treatment increased the release of ROS. AK020546 overexpression reduced this elevation while miR-350-3p reversed the effect of AK020546 (Figure 6J).

### MiR-350-3p directly targeted ErbB3 in H9c2 cardiomyocytes

**Figure 7:**
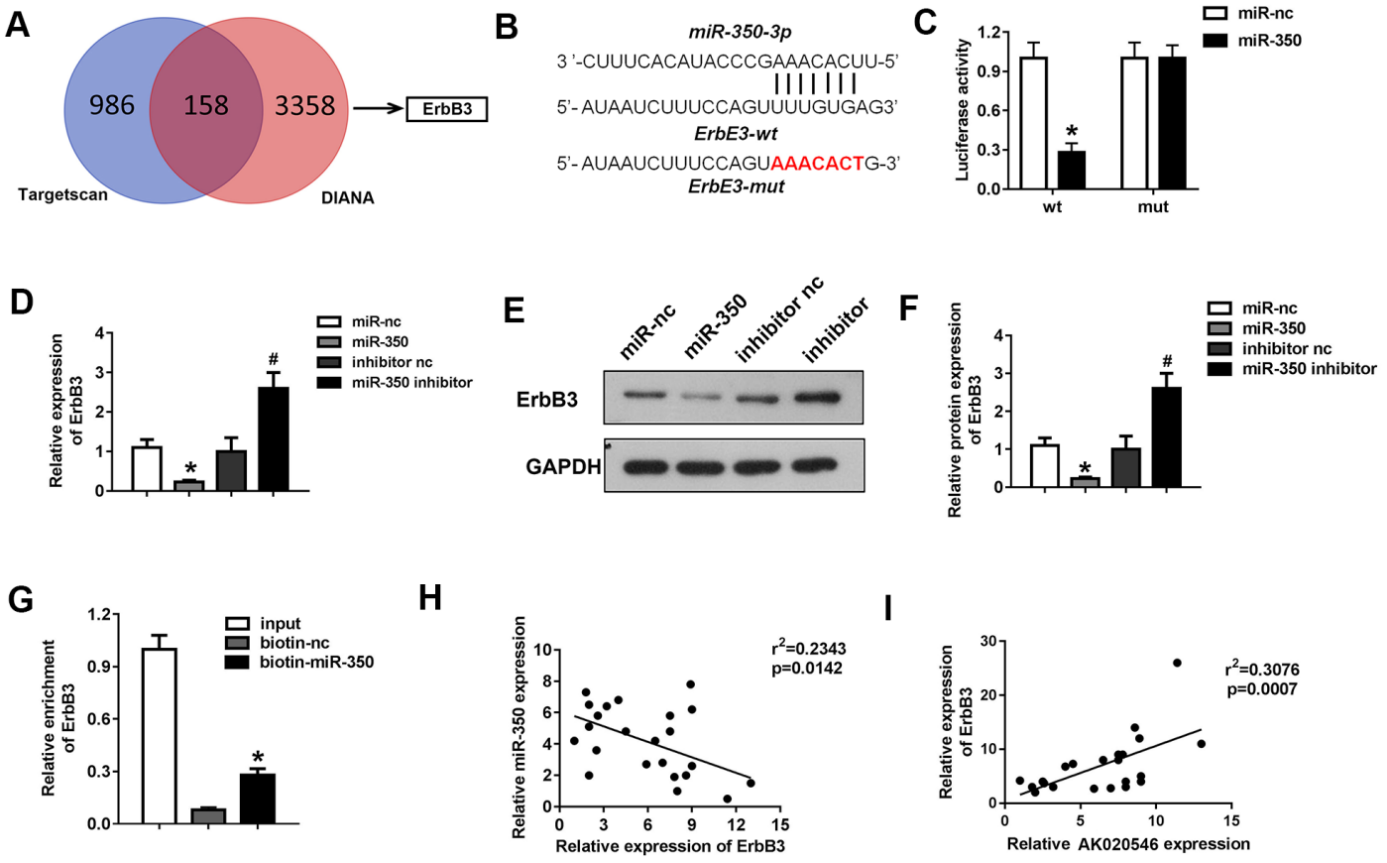
**MiR-350-3p directly targeted ErbB3 in H9c2 cardiomyocytes.** (**A**) Bioinformatics analysis using TargetScan and DIANA predicted ErbB3 as the target of miR-350-3p. (**B**) The potential targeting region between miR-350-3p and ErbB3 predicted by bioinformatics analysis. (**C**) Luciferase assay was performed to verify whether miR-350-3p targeted ErbB3 in H9c2 cardiomyocytes (*n* = 5). (**D**) qPCR was used to detect the expression of ErbB3. (**E**, **F**) Western blot was carried out to evaluate the expression level of ErbB3 (*n* = 5). (**G**) Luciferase assay was performed to verify whether miR-350-3p targeted ErbB3 in H9c2 cardiomyocytes (*n* = 5). (**H**, **I**) Pearson’s analysis was performed to investigate the correlation between AK020546 and ErbB3 as well as miR-350-3p and ErbB3 (*n* = 5). ^*^p < 0.05 vs miR-nc or biotin-nc, ^#^*p* < 0.05 vs inhibitor nc group.

DIANA and TargetScan tools were used to predict the downstream target genes of miR-350-3p. ErbB3 was predicted as a potential target by both datasets (Figure 7A). Figure 7B shows the target region between miR-350-3p and ErbB3. Next, luciferase assay in H9c2 cardiomyocytes demonstrated that miR-350-3p downregulated the luciferase activity in cells co-transfected with pGL3-3’UTR of ErbB3, but not the pGL3-3’UTR-mut of ErbB3 (Figure 7C). Further, qPCR results revealed that miR-350-3p overexpression decreased the mRNA expression of ErbB3, whereas its knockdown increased the expression (Figure 7D). Western blotting results indicated that miR-350-3p overexpression decreased the protein level of ErbB3, whereas its knockdown reversed this effect (Figure 7E, 7F). Moreover, RNA pull-down assay confirmed that miR-350-3p directly bound to ErbB3 (Figure 7G). Finally, Pearson’s analysis revealed a negative correlation between miR-350-3p and ErbB3 and a negative correlation between AK020546 and miR-350-3p. These results indicated that lncRNA AK020546 functioned as a ceRNA for miR-350-3p and activated its target gene *ErbB3* (Figure 7H, 7I).

### LncRNA AK020546 increased the expression of *ErbB3* and activated the downstream AKT pathway

**Figure 8:**
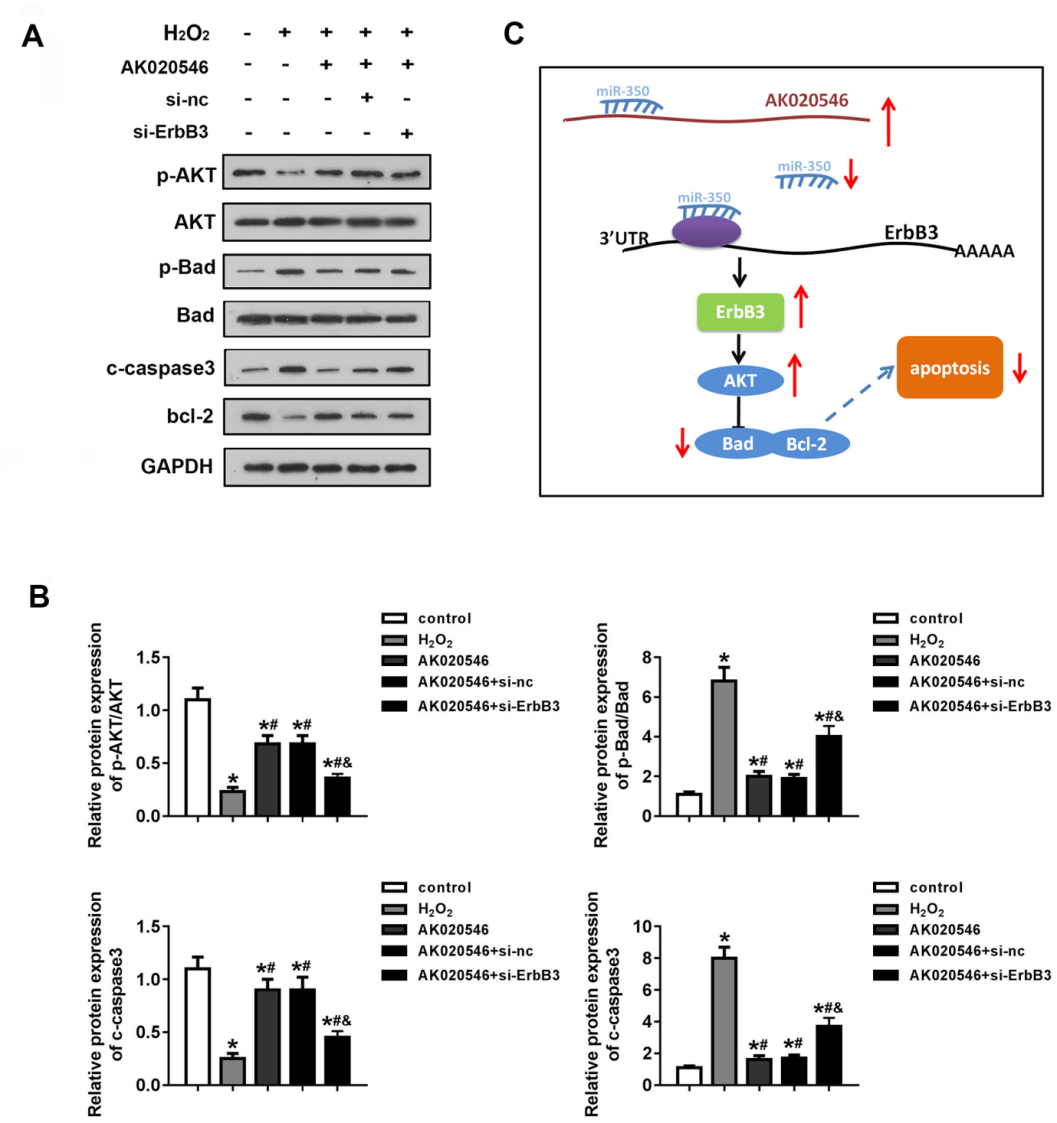
**LncRNA AK020546 activated the AKT signaling pathway.** (**A**, **B**) Western blotting was used to evaluate the expression of AKT, Bad, c-Caspase 3, Bcl-2, p-AKT, and p-Bad (*n* = 5). (**C**) Flow diagram representing the molecular mechanism. ^*^p < 0.05 vs control, ^#^*p* < 0.05 vs H_2_O_2_ group, ^&^*p* < 0.05 vs AK020546 + *si-ErbB3* group.

ErbB3 is involved in cell apoptosis and oxidative stress during I/R injury. For instance, ErbB3 was reported to regulate the phosphorylation of AKT and Bad that are involved in cardiac I/R injury. We further evaluated the expression of apoptotic proteins such as Bcl-2, Bax, Bad, and cleaved Caspase 3 (c-Caspase 3). AK020546 elevated the expression of ErbB3, Bcl-2, pAKT, whereas it attenuated the expression of c-Caspase 3 and the phosphorylation of Bad (Figure 8A, 8B). Figure 8C shows the proposed mechanism of the effect of lncRNA AK020546.

## DISCUSSION

LncRNAs have been implicated in several physiological and pathological processes such as cell proliferation, differentiation, development, and apoptosis. Moreover, lncRNAs can function as competing endogenous RNAs (ceRNAs) that sequester miRNAs to regulate the expression of miRNA target genes. For instance, lncRNAs such as APF, MALAT1, and PFL participate in I/R injury by sponging miRNAs [22–25]. LncRNA AK020546, also known as CAIF, has been implicated in several cellular processes. For instance, AK020546 is downregulated in osteoarthritis and its overexpression improves the condition by sponging miR-1246 [26]. Zhou et al. reported that AK020546 participated in myocardial remodeling [27]. Similarly, Wu et al. reported MALAT1 to be a promising diagnostic and prognostic marker in end-stage cardiomyopathy. AK020546 has been reported to inhibit autophagy and attenuate acute myocardial infarction via the p53–myocardin axis [27]. We demonstrated that AK020546 was downregulated in AMI both *in vivo* and *in vitro*. Its overexpression reduced the infarct area and restored heart function. Moreover, AK020546 overexpression inhibited the release of ROS and apoptosis of H9c2 cardiomyocytes, implying its cardioprotective function in AMI.

Bioinformatic tools predicted miR-350-3p as a potential target of AK020546, which was experimentally confirmed by luciferase assay. MiR-350-3p promotes RAW264.7 cell apoptosis by repressing the PIK3R3/MAPK signaling pathway [28]. In addition, a study reported miR-350 as a promising biomarker of smoking-related interstitial fibrosis (SRIF) [29]. Another study reported elevated expression of miR-350 in late-stage transverse aortic constriction (TAC) in rat heart. Moreover, miR-350-3p contributes to pathological cardiac hypertrophy and apoptosis by inactivating the p38/JNK pathway [14, 15]. These findings demonstrated the function of miR-350-3p in cardiovascular diseases. Considering the relationship between myocardial hypertrophy and myocardial ischemic injury, we hypothesized that miR-350-3p exerted a substantial effect during I/R injury. We confirmed miR-350-3p as a target of AK020546 and that AK020546 exerted its cardioprotective function via sponging miR-350-3p. Overexpression of miR-350-3p reversed the effects of AK020546 on I/R injury including ROS release and apoptosis.

ErbB3 belongs to the PA2G4 family and participates in multiple cellular processes including cell cycle, proliferation, and survival [30–34]. Several studies have reported the involvement of ErbB3 in cardiovascular diseases. For instance, methylation of the *ErbB3* gene has been observed in human dilated cardiomyopathy. In addition, ErbB3 degradation by Nrdp1, an E3 ligase, following I/R injury in cardiomyocytes promotes apoptosis and alleviates I/R injury [21, 35]. ErbB3 activates several protein kinases involved in cell apoptosis, including AKT, STAT3, and mitogen-activated protein kinases (MAPKs) [36–38]. The PI3K/AKT pathway is triggered in response to I/R injury and promotes cell survival by inhibiting caspase-induced apoptosis [39, 40]. We found that AK020546 elevated the expression of Bcl-2 and phosphorylation of AKT, whereas decreased the protein levels of c-Caspase 3 and phosphorylation of Bad; these effects were reversed by *ErbB3* knockdown. We speculated that AK020546 sponged miR-350-3p and blocked the inhibitory effect of miR-350-3p on *ErbB3* expression to promote AKT phosphorylation and the expression of anti-apoptotic protein Bcl-2, consequently decreasing cell apoptosis in H9c2 cardiomyocytes.

Our study had certain limitations. Several studies have implicated altered mitochondrial functions, including increased mitochondrial membrane depolarization [41, 42] and disrupted activity of enzymes of the electron transport chain [43], in cardiac I/R injury. However, our study lacked data on the involvement of mitochondrial dysfunction. Furthermore, we did not study the function of gap junction protein Cx43, increased expression of which is associated with reduced reperfusion arrhythmia in cardiac IR injury [43–45].

In conclusion, lncRNA AK020546 was downregulated in AMI and its overexpression restored heart function, suppressed ROS release, and reduced the apoptosis of H9c2 cardiomyocytes. LncRNA AK020546 exerted these functions by activating ErbB3 via sponging miR-350-3p.

## MATERIALS AND METHODS

### Animals

Wistar rats were purchased from Charles River Laboratories (Beijing, China). These rats were housed in a environment of specific pathogen-free (SPF) and are with ad lib access to water and food. All *in vivo* studies were approved by the Animal Studies Committee of Zhejiang Renmin Hospital and were performed following the institutional guidelines.

### Plasmid construction and transfection

MiR-350-3p mimic, siRNA against ErbB3, adenovirus overexpressing AK020546 along with their controls were purchased from GenePharma (Shanghai, China). The wild type and mutant type of binding cites among AK020546 and ErbB3 sequence were synthesized by GenePharma followed by cloning into the pGL3 reporter vector (Promega, CA, USA).

### I/R model establishment

All rats were kept on a 12-h light/dark cycle and in an environment of 25 and 60% humidity with ad lib access to water and food. 32 rats were randomly divided into 4 different groups: sham group, I/R group, IR + Ad-nc group, and IR + Ad-AK020546 group. Afterward, the rats were administered of Ad-AK020546 or Ad-nc at the dose of 1 × 109 plaque-forming units (PFUs) for 3 consecutive days via tail vein, whereas the rat in sham control group was injected with saline. Four days later, the I/R injury was induced in rats as per the method described previously [46]. Briefly, rats were intraperitoneally injected with 1% pentobarbital sodium (50 mg/kg; Sigma-Aldrich, P3761, USA). After thoracotomy, the intercostal muscle was separated in the 3rd and 4th intercostal space to expose the heart. The middle of the left anterior descending coronary artery was ligated using a 6-0 surgical suture. The sham rats underwent surgery but without ligation of left anterior descending (LAD) coronary artery ligated using a 6-0 silk suture. The I/R procedure included a 30 min of ischemia and 2 h of reperfusion. All rats were sacrificed immediately after anesthetization with 3% pentobarbital sodium (160 mg/kg). Subsequently, heart tissue and blood samples were collected for further experiments following I/R injury.

### Echocardiography

Transthoracic echocardiography detection was carried out with a VisualSonics Vevo 2100 (VisualSonics) ultrasound system and a 40-MHz transducer. A 1.4-F Millar catheter (Millar Instruments, UT, USA) was inserted into the carotid artery of mice, and then left ventricular end-diastolic pressure (LVEDP) left ventricular systolic pressure (LVSP), the maximal rate of LV contractility (+dp/dt), and the maximal rate of LV relaxation (−dp/dt) were obtained using the ultrasound device as described previously [47].

### TTC staining

For TTC staining, 2% Evans blue was injected into the heart from the femoral vein after the ligation of LAD. Heart tissues were collected immediately after the injection and rinsed with ice–cold normal saline (NS). The samples were frozen at –20° C for 30 min and subsequently transversely cut into 1 mm-thick slices. These tissues were incubated with TTC at 37° C for 15 min. After fixation with formaldehyde for 24 h, the samples were visualized and photographed.

### Cell culture

H9c2 cardiomyocytes were provided by the Cell Bank of Chinese Academy of Sciences. Cells were maintained in DMEM (Gibco, USA) containing 10% fetal bovine serum (FBS, Gibco, USA) and supplemented with 100 U/mL penicillin, 100 μg/mL streptomycin, and 110 mg/mL sodium pyruvate at 37° C under a humidified conditions of 95% air and 5% CO2. For the establishment of oxidative injury model, H9c2 cells were cultured with 600 μM of H_2_O_2_.

### TUNEL staining

The TUNEL staining was carried out using an *in situ* Cell Death Detection Kit (Roche, Mannheim, Germany). After fixation and dehydration, the heart tissues were embedded in paraffin followed by cutting into 4 μm sections. The experimental procedures were performed as per the manufacturer’s instructions. Slices were mounted and imaged under a fluorescence microscope (Nikon, Japan). Subsequently, the ratio of apoptotic cells to total cardiomyocytes was calculated.

### Flow cytometry

Approximately 1× 106 /mL H9c2 cells were collected, centrifuged at 500-1000 r/min for 5 min. After washing with 3 ml PBS and another centrifugation (500-1000 r/min for 5 min), the cells were fixed with precooled 70% ethanol for 2 h at 4° C. Then, the cells were incubated with FITC- AnnexinV (300ng/mL, 4° C) for 10 min to label apoptotic cells. The samples were further incubated with propidium iodide (PI) for 5 min. The apoptotic cells were then detected with a Fortessa flow-cytometer system (Becton Dickinson, NJ, USA).

### JC-1 staining

JC-1 (10 mM, Beyotime, China) was applied to incubate with H9c2 cells at 37° C for 30 min in dark condition. Then, the H9c2 cells were rinsed and resuspended with PBS and imaged under a fluorescence microscope (Nikon, Japan). When the level of JC-1 is reduced, green fluorescence could be observed. Green fluorescence indicates the mitochondrial inner membrane potential is disrupted.

### Malondialdehyde (MDA) and lactate dehydrogenase (LDH) evaluation

The heart tissues were grind with lysis buffer and centrifuged at 1,500 g for 15 min at 4° C. After the supernatant was extracted, the amount of LDH and MDA in the supernatant was detected using commercial kits following the manufacture’s protocols at -20° C (Beyotime).

### ROS detection

2’,7’-dichlorofluorescin diacetate (DCFH) (Jiancheng, Nanjing, China) was applied to assess the level of ROS. Briefly, after H/R or I/R treatment, cells or myocardial samples were rinsed with PBS (4° C). Then, the samples were incubated with DCFH (10 μM) at 37° C for 20 min in dark incubator. The stained samples were photographed under a fluorescence microscope (Nikon, Japan) at 488 nm excitation and 590 nm emission, respectively. The mean fluorescence intensity (MFI) was analyzed using ImageQuant version 5.2.

### Mitochondrial ROS detection

Mitochondrial ROS was assessed using a MitoSOX kit (Thermo Fisher Scientific, M36008, USA) following manufacturer's protocols. After transfection and treatment, MitoSOX was added to the H9c2 cells and incubated at 37° C for 20 min. The relative fluorescence intensity was calculated in different groups.

### Quantitative real-time PCR (qPCR)

The RNA was extracted using TRIzol reagent (Invitrogen, USA) following the manufacturer’s protocols. 1 μg RNA was reverse transcribed into cDNA using a Reverse transcription kit (TransGen, Beijing, China). Thereafter, 10 ng cDNA was used for qPCR experiments as the template. The relative expression of genes was evaluated using a SYBR green mix (Yisheng, Shanghai) in a 7500 Fast Realtime PCR system (Applied Biosystems, USA). The sequences of primers used in this study are as follows: miR-350-3p forward: 5’-TGCGGTTCACAAAGCCCATAGAG-3’ and reverse: 5’-CCAGTGCAGGGTCCGAGGT-3’; lncRNA AK020546 forward: 5’-TCGTGAATTTTGTCAGTTTTGTGATATCC-3’ and reverse: 5’-AGGTAAGTTTAACTGGTCAGGAAATAAAC-3’; BbrB3 forward: 5’-CGTCATGCCAGATACACACC-3’ and reverse: 5’-CTCCTCGTACCCTTGCTCAG-3’; U6 forward: 5’-CTCGCTTCGGCAGCACA-3’ and reverse, 5’-TAGTCCTTCCTACCCCAATTTCC-3’; GAPDH forward: 5’-TCATGACAACTTTGGCATCATGG-3’ and reverse: 5’-GTCTCCTGACTTCAACAGCAAC-3.’

### Western blotting

Total proteins were extracted with RIPA buffer containing protease inhibitor and quantified with a BCA protein analysis kit (Beyotime, Shanghai, China). Next, 20 μg of protein was separated by 10% SDS-PAGE (Thermo Fisher Scientific, Inc., Waltham, USA) and transferred to PVDF membranes. The membranes were incubated in 5% non-fat milk at room temperature for 2 h. Afterward, the membranes were incubated at 4° C overnight in dark with the following primary antibodies: anti-ErbB3 (1:1000; ab32121; Abcam, Shanghai, China), anti-p-AKT (1:1000; ab38449), anti-AKT (1:1000; ab179463; Abcam), anti-Bad (1:2000; ab32445; Abcam), anti-p-Bad (1:5000; ab129192; Abcam), anti-cleaved Caspase 3 (1:500; ab32042; Abcam), anti-Bcl-2 (1:1000; ab32124; Abcam), and anti-GAPDH (1:2500; ab9485; Abcam). Next, after washing with Tris/Tween-20 for 3 times, the blots were incubated with horseradish peroxidase (HRP)-conjugated secondary antibody (1:1000; ab7090; Abcam) at room temperature for another 2 h. Finally, the proteins were visualized by BeyoECL Plus Kit (Beyotime) and quantified with the ImageJ software (version 1.8.0, National Institutes of Health, USA).

### Pull-down assay

The biotin-labeled miR-350-3p probe and the control were purchased from Sangon Biotech (Shanghai, China). These probes were incubated with the streptavidin-coated beads (Invitrogen) at 25° C for 2 h. Thereafter, H9c2 cells were lysed, and the biotin labeled probes were added into the lysate. The incubation last overnight at 4° C. Subsequently, the bound RNAs were eluted from the beads and purified using with TRIzol reagent (Takara, China). Finally, the enrichment of AK020546 or ErbB3 in the complex was evaluated by qPCR as mentioned above.

### Luciferase assay

The wild or mutant type of binding sites among AK020546 or ErbB3 sequence were cloned into pGL3 luciferase reporter vector. H9c2 cells were co-transfected with miR-350-3p mimic or mimic control along with the reporters carrying those wild type or mutant binding citesTRL-SV40 vector served as internal control (Promega, USA) was transfected. 48 h later, the H9c2 cells were collected followed by detecting the luciferase activity with a dual-luciferase assay kit (Promega, USA).

### Statistical analysis

All data was presented as the means ± standard deviations (SDs) of at least three independent experiments. Statistical analysis was performed with the SPSS 17.0 (SPSS, Inc., IL, USA). One-way analysis of variance (ANOVA) followed by Tukey’s post-hoc test was performed to evaluate the difference between multiple groups. *p*< 0.05 was considered as statistical significant.
